# Formic Acid-Based Preparation in *Varroa destructor* Control and Its Effects on Hygienic Behavior of *Apis mellifera*

**DOI:** 10.3390/insects16121236

**Published:** 2025-12-06

**Authors:** Marko Ristanić, Uroš Glavinić, Jevrosima Stevanović, Tamara Cvetković, Aleksa Mijatović, Branislav Vejnović, Zoran Stanimirović

**Affiliations:** 1 Department of Biology, Faculty of Veterinary Medicine, University of Belgrade, 11000 Belgrade, Serbia; mristanic@vet.bg.ac.rs (M.R.); rocky@vet.bg.ac.rs (J.S.); aleksa.mijatovic@vet.bg.ac.rs (A.M.); zoran@vet.bg.ac.rs (Z.S.); 2 Department of Economics and Statistics, Faculty of Veterinary Medicine, University of Belgrade, 11000 Belgrade, Serbia; branislavv@vet.bg.ac.rs

**Keywords:** formic acid, Formic Pro™, *Varroa destructor*, hygienic behavior

## Abstract

*Varroa destructor* remains a major threat to honey bee (*Apis mellifera*) health, requiring effective and sustainable control strategies. In this study, a formic acid-based product (Formic Pro™) was tested for its varroacidal efficacy and effects on hygienic behavior of honey bee colonies. Sixty hives were assigned to three groups: Formic Pro group (*n* = 30), Positive control treated with amitraz (*n* = 15), and untreated Negative control (*n* = 15). Efficacy was assessed by daily mite drop counts, while hygienic behavior was evaluated before and after treatment using the “pin-killed” brood method. The Formic Pro group achieved a mite mortality rate of 88.37% ± 0.23, while amitraz as a control exerted 94.30% ± 0.95 efficacy. The treatment was well tolerated, with no queen losses, minimal and transient brood effects, followed by brief bearding on the first day of trial. Hygienic behavior significantly increased in the Formic Pro group (PCC from 96.69% to 99.01%, *p* < 0.001), while it declined in the Negative control and remained unchanged in the Positive control group. These results demonstrate that the tested formic acid-based product provides high acaricidal efficacy which is tolerated well in colonies and that it stimulates a key behavioral defense mechanism through hygienic behavior, providing benefits both for *Varroa* control and colony resilience.

## Simple Summary

*Varroa destructor* presents one of the leading parasites of honey bees. Controlling the loads of this ectoparasite in hives is crucial in order to maintain healthy colonies and effective pollination. This study investigated the efficacy of a formic acid-based product (Formic Pro™) in field conditions. It achieved varroacidal efficacy of 88.37%, without causing queen losses or significant brood disruption. Furthermore, a key natural defense mechanism presented through the hygienic behavior of worker bees was significantly stimulated. Our findings indicate that the tested product Formic Pro™ can both control *Varroa* populations and support colony health, making it a valuable component of integrated pest management strategies for sustainable beekeeping.

## 1. Introduction

The honey bee mite *Varroa destructor* is one of the most critical biological threats to honey bees (*Apis mellifera*) worldwide [1]. Since its host switch from *Apis cerana* to *A. mellifera*, this parasite has spread globally, causing significant colony losses and impacting pollination [2]. Not only does *Varroa* feed on the fat body of adult and developing bees but it is also a vector of numerous honey bee viruses, including Deformed Wing Virus and Acute Bee Paralysis Virus, adding to the destructive effects of this mite [3,4,5,6].

Standard *Varroa* management has relied on synthetic acaricides such as coumaphos, fluvalinate, and amitraz. However, over time, widespread resistance to these “hard acaricides” has been noted [7,8], making it crucial to investigate alternative control strategies. Organic acids, such as formic acid, have emerged as a promising solution due to their lack of persistent residues, compatibility with organic beekeeping, and unique ability to penetrate sealed brood cells, unlike most contact preparations [9,10,11].

Several delivery methods for formic acid have been studied over the years, from absorbent pads and gel formulations to commercial slow-release products. Among them, the commercial product Formic Pro™ (NOD Apiary Ireland Ltd., Listowel, Ireland) has gained attention for its standardized release mechanism and reduced brood and queen toxicity risk [12,13]. This commercial product contains 68.2 g of formic acid per strip and is designed to deliver a steady dose under proper environmental conditions. Formic Pro™ employs a polymer-stabilized gel matrix designed to regulate the evaporation kinetics of formic acid. This matrix limits the exposed surface area of the acid and buffers against rapid phase transitions, resulting in a controlled and predictable release rate. Under the recommended application temperature range (approximately 10–29 °C), the gel ensures gradual volatilization resulting in lower peak concentrations compared to liquid formulations. By stabilizing the evaporation process and minimizing temperature-induced fluctuations, the product achieves a standardized release profile that allows reliable field performance across diverse environmental conditions. Even though internationally validated, there is a need to test such treatments under specific local climates, beekeeping practices, and colony development dynamics. In order to comprehend the full effect of formic acid on the colony, certain parameters such as the hygienic behavior of worker bees, queen activity, and health are often monitored together with anti-varroa efficacy.

The hygienic behavior of honey bees serves as an indicator of their ability to resist diseases and parasites, particularly those affecting the brood, such as American foulbrood, chalkbrood, and *Varroa destructor* mites [14]. Consequently, the level of hygienic behavior is commonly assessed in honey bee breeding programs [15,16]. Nevertheless, hygienic behavior as a highly polygenic trait [17] can be influenced by external factors, either negatively, as shown for pesticides, imidacloprid and clothianidin [18,19], and coumaphos [20], or positively, as achieved by thymol [20,21] and commercial diet supplements [22,23]. Recent studies further highlight that the interaction between honey bees and *Varroa* mites is shaped by multiple additive mechanisms, including immunological, physiological, and behavioral adaptations that affect colony-level tolerance to infestation [24]. Moreover, recent field trials demonstrate that nutritional interventions can significantly enhance hygienic and grooming behavior, thereby improving colony resilience under natural infestation pressure [23]. For the products used in this study, formic acid and amitraz, there are reports of negative effects (formic acid: brood, queen, and drone mortality; larval and pupal tissue damage; colony weakening; triggering of physiological stress responses; amitraz: physiological impairment of workers; impaired immune function; drone reproductive dysfunction; brood development disruption; production of persistent toxic metabolites) on honey bees [25], but not on the hygienic behavior of bees [20]. Considering the aforementioned, this study aims to evaluate the effectiveness and safety of Formic Pro™ under field (bee hive) conditions, as well as its effect on the hygienic behavior of honey bees, in order to propose recommendations for integrated pest management (IPM) approaches in Serbia.

## 2. Materials and Methods

### 2.1. Study Site, Climatic Conditions, and Honey Bees

In our investigation during August 2023, we tested the efficacy of the formic acid-based product Formic Pro™ (NOD Apiary Ireland Ltd., Listowel, Ireland) on *Apis mellifera carnica* colonies located in the urban research apiary of the Department of Biology, Faculty of Veterinary Medicine, University of Belgrade (44.793877 N; 20.464101 E). The Belgrade area has a warm-temperate continental climate, with hot summers and relatively mild autumns. According to local meteorological records, the average daily air temperature in August–September 2023 ranged between approximately 22 and 24 °C, with mean daily maxima most often between 27 and 32 °C and minima between 15 and 20 °C. During the 15-day Formic Pro^®^ treatment period, daytime temperatures generally remained within this range and did not exceed the upper safety limits recommended for formic acid application, providing conditions typical for the late-summer period in the region. The colonies were equalized in respect of their strength parameters (open brood area, sealed brood area, reserves of stored honey and pollen/bee bread, and adult bee population) according to convenient beekeeping practice [26,27] and the Liebefeld method [28]. Before the start of the experiment, all hives had two brood chambers, both with nine intercomb spaces (all “bee spaces” occupied by bees) and with three food store frames (honey and bee bread). Colonies were regularly checked for both bee and brood pathology by a veterinary specialist (one of the authors of this work), and no disease, except varroosis, was affirmed. The experiment was conducted on 60 Langstroth-Root (LR) hives composed of two chambers, equipped with screened bottom boards that remained fully open throughout the experiment, as well as fully open hive entrances. The bottom boards had metal inserts with white cardboard covered with a thin layer of adhesive material (edible sunflower oil) to keep the mites on the insert until they were counted and thus ensure the reliable evaluation of the number of knocked-down mites and the varroacidal efficacy of the test product [22,29]. The described hive configuration ensured strong natural vertical ventilation and reflected routine local beekeeping practice during the late-summer nectar flow. No additional mechanical ventilation or insulation was used beyond the standard hive design.

### 2.2. Varroacidal Efficacy Assessment

**Figure 1:**
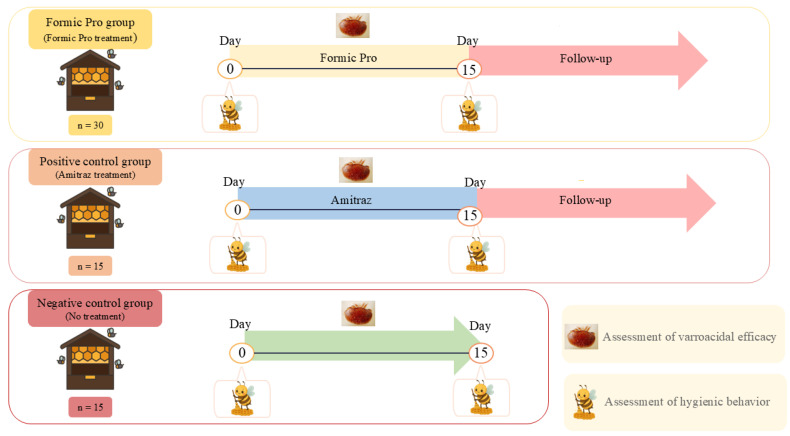
Experimental design showing the timeline of treatments, assessment of varroacidal efficacy and time-points of hygienic behavior evaluation in all groups.

Thirty hives were assigned to the Formic Pro group and treated with Formic Pro™, 15 hives to the Positive control group, treated with amitraz (CAS No.: 33089-61-1, applied by fumigation as described in Stanimirovic et al. [29]), and 15 hives to the Negative control group, left untreated (Figure 1). The initial *Varroa* infestation was estimated using natural mite fall measured over two days before treatment, yielding an average of 6.36 mites per colony per day. Using standard conversion factors (120–200 × daily natural mortality), the initial colony infestation was estimated at approximately 750–1300 mites per colony. This is within the European Medicines Agency (EMA) [30] recommended range of 300–3000 mites for field efficacy trials.

Treatment with the test product (Formic Pro™) lasted 14 days, following the manufacturer’s instructions. Two strips of Formic Pro™ (each containing 68.2 g formic acid) were placed on the top bars of the frames in each treated hive of the Formic Pro group. The efficacy of the product was evaluated by daily counting the fallen *Varroa* mites on the metal insert of the treatment hives’ bottom boards. The obtained results were used for the evaluation of Formic Pro™ efficacy compared to amitraz and using oxalic acid as a follow-up treatment for both (Formic Pro group and Positive control–amitraz group), having in mind that it is highly (>95%) efficient [31], following the recommendation given by EMA [30]. Oxalic acid (CAS No.: 144-62-7) was applied in the form of strips (“GB Strong”, Golden Bee d.o.o., Belgrade, Serbia). Mite mortality was calculated using the standard formula: Mite Mortality (%) = Number of mites fallen during treatment period/Total mites (number of mites fallen after test treatment and follow-up treatment × 100) [32,33].

### 2.3. Hygienic Behavior Evaluation

Hygienic behavior was evaluated by the “pin-killed” brood assay, where only fully capped worker pupae at the red-eye to dark-eye developmental stage (approximately 9–12 days post-capping) were used, following established methodological recommendations [15,34,35,36]. The evaluation was performed before and after the 15-day treatment period (on day 0 and day 15 in each group). Briefly, from each colony, we chose one frame with a continuous, fully capped worker brood on both sides. A parallelogram-shaped wire template (5 × 6 cm) was placed on the brood, and then all pupae within the template were counted and killed with an entomological pin. After completing this procedure, the frame was marked and returned to the colony. After 24 h, the “pin-killed” areas were checked, and the cells that had been completely cleaned were counted. The assay scores are calculated as the percent of cleaned cells (PCC) and represent the level of hygienic behavior. Furthermore, if more than 95% of the pin-killed cells were cleaned, the colony was considered super-hygienic; if the efficiency of pupae removal was between 90% and 95%, the colony was proclaimed hygienic, while non-hygienic colonies were those which cleaned less than 90% of the sacrificed brood [36].

### 2.4. Statistical Analyses

The data obtained for varroacidal efficiency and hygienic behavior were tested for normality using the Shapiro–Wilk test. As the data followed a normal distribution (Shapiro–Wilk test, *p* > 0.05), differences in mean values for varroacidal efficiency between groups were assessed using a one-way ANOVA, followed by Tukey’s post hoc test. For hygienic behavior, group comparisons were performed using a two-way ANOVA with repeated measures on one factor, followed by Tukey’s test for within-group comparisons and Sidak’s test for between-group comparisons over time. The results are presented as mean ± standard deviation (x¯ ± SD). Significant difference was estimated at *p* < 0.05, *p* < 0.01, and *p* < 0.001 significance levels. Statistical analysis of the results obtained in the experiment was carried out using statistical software GraphPad Prism version 7 (GraphPad, San Diego, CA, USA).

## 3. Results

### 3.1. Varroacidal Efficacy

**Figure 2:**
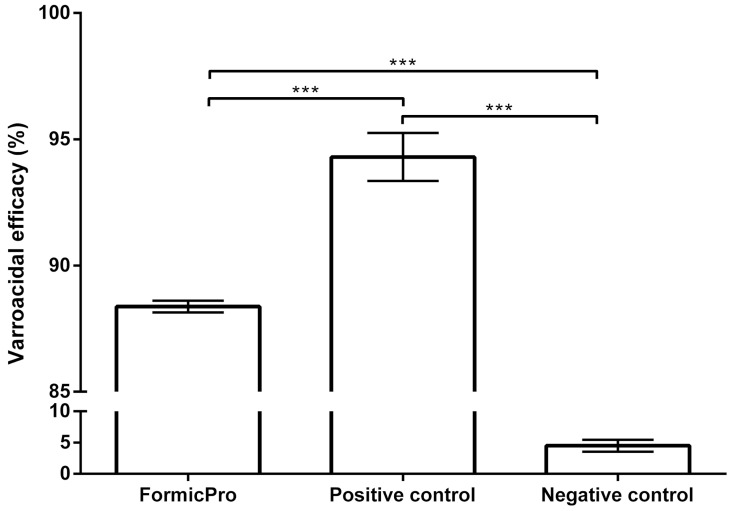
Varroacidal efficacy of the tested product Formic Pro™ in relation to the Positive and Negative control. *** *p* < 0.001.

Applying the formula recommended by EMA [30], the efficacy of Formic Pro™ and amitraz as a Positive control, both followed up with oxalic acid treatment, was determined. The effectiveness of the test product (Formic Pro™) was 88.37% ± 0.23 (Table 1; Supplementary Table S1) while the efficacy of amitraz was 94.30% ± 0.95 (Figure 2).

Due to the normal distribution of the results obtained using the Shapiro–Wilk test, we examined the significance of differences between groups. The applied analyses determined the existence of a high degree of significance (*p* < 0.001) among the compared groups (Figure 2). Application of the tested product on the 1st day resulted in bearding (a phenomenon where bees accumulate around the hive entrance in a beard-like shape) in six hives, while the others showed greater anxiety. On the same day, in the evening hours, bearding was not observed. During and after the end of the treatment, no loss of queens was recorded, while brood disruption was minimal and temporary, aligning with the expected biological effects of formic acid. At the end of the entire treatment cycle and the application of the follow-up treatment, a significant intake of nectar was observed, and honey chambers were full (20–25 kg of honey), which necessitated the addition of a third honey chamber on all hives.

### 3.2. Hygienic Behavior

Indicators of descriptive statistics for hygienic behavior (expressed through PCC) for each group, before treatment (Day 0) and after the treatment period (Day 15), are shown in Table 2.

**Figure 3:**
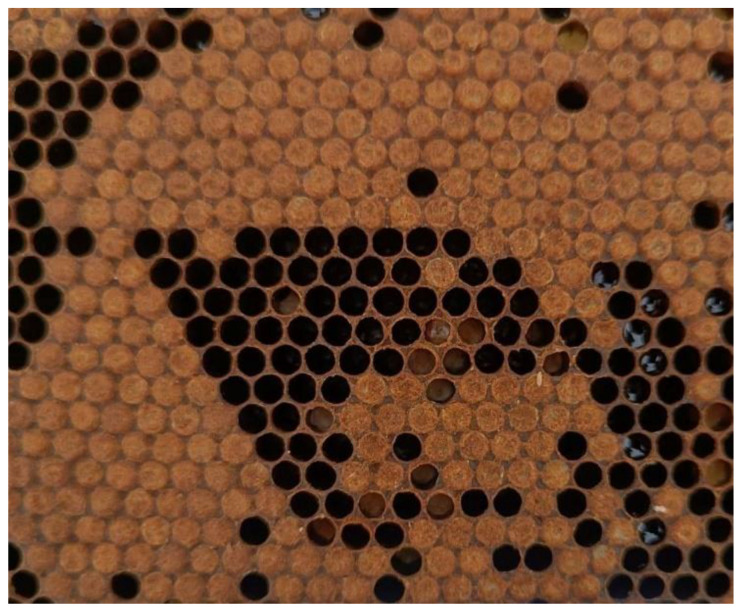
Non-hygienic behavior (<90% of cleaned cells after 24 h) of honey bee colony in Negative control group.

The average PCC in the experimental groups before the treatment period ranged from 96.69% to 98.10%, and shifted to a range of 90.72% to 99.01% after the treatment period. The lowest PCC following the treatment period was recorded in the Negative control group, with individual values dropping to 86.36% and a group average of 90.72%, indicating an apparent decline in hygienic behavior (Figure 3).

**Figure 4:**
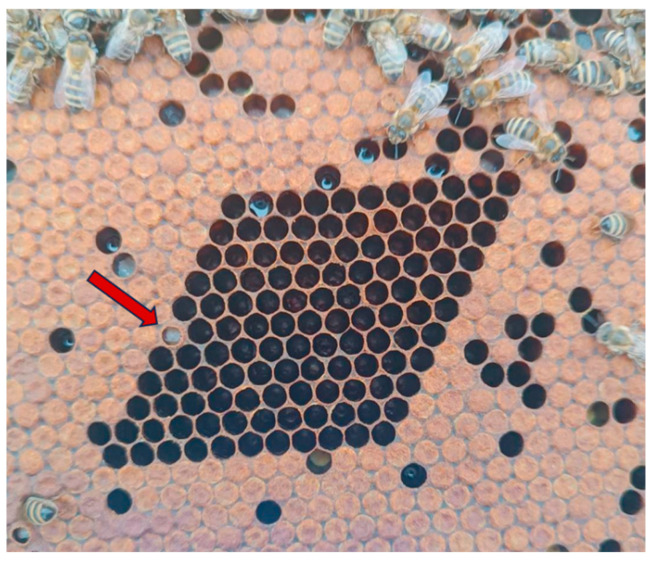
Super-hygienic behavior (100% of cleaned cells after 24 h) of honey bee colony in Formic Pro group. Red arrow indicates control non-killed pupae.

In contrast, the Formic Pro group exhibited the highest post-treatment PCC, reaching a value up to 99.45%, with an average value of 99.01%, indicating an intense stimulation of hygienic behavior (Figure 4).

In the Positive control group (amitraz-treated), PCC remained almost unchanged, suggesting no significant effect of the treatment (*p* > 0.05) on bee hygienic behavior (Figure 4).

**Figure 5:**
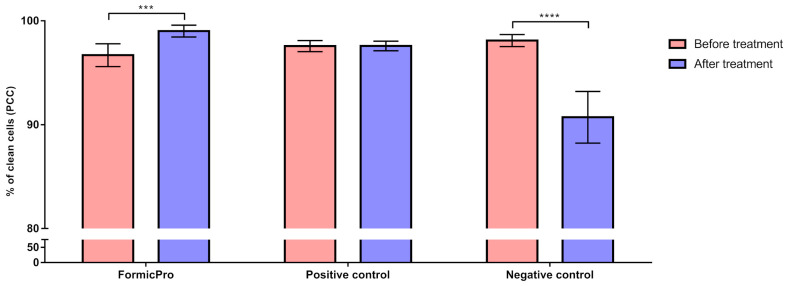
Comparison of hygienic behavior values in each experimental group (Formic Pro, Positive control, and Negative control) before and after the 15-day treatment period. *** *p* < 0.001; **** *p* < 0.0001. The error bar stands for standard deviation.

The results of the two-way ANOVA with repeated measures on one factor, followed by Sidak’s post hoc test, showed that the level of hygienic behavior recorded after the treatment period was significantly different than that before the treatment period in the Formic Pro group and the Negative control, with PCC significantly (*p* < 0.001) increasing in the Formic Pro group, and PCC significantly (*p* < 0.0001) decreasing in the Negative control (Figure 5).

**Figure 6:**
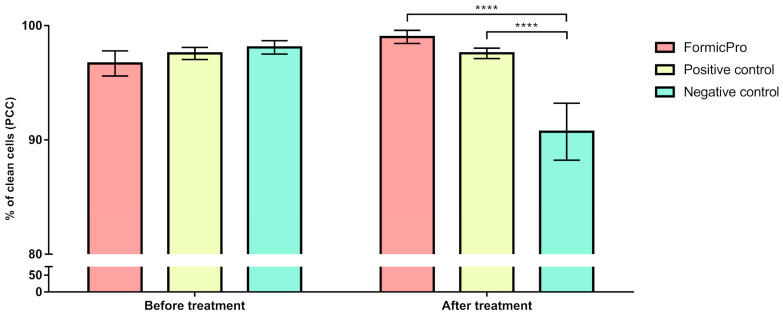
Comparison of hygienic behavior values in different occasions (before and after the 15-day treatment period) between Formic Pro, Positive control, and Negative control groups. **** *p* < 0.0001. The error bar stands for standard deviation.

The results of the two-way ANOVA with repeated measures on one factor, followed by Tukey’s post hoc test, indicated no significant differences in hygienic behavior levels between groups before the treatment period. However, after the treatment period, PCC values in the Formic Pro and Positive control groups were significantly (*p* < 0.0001) higher than in the Negative control group (Figure 6).

## 4. Discussion

The present study demonstrated that the formic acid-based product Formic Pro™ achieved 88.37% ± 0.23 efficacy against *Varroa destructor*, compared to amitraz, which exerted high (94.30% ± 0.95) efficacy. Our findings are in accordance with earlier studies of the efficacy of different formulations based on formic acid which consistently describe formic acid as an effective ecological treatment available in *Varroa* management [12,33,37,38]. In our trial, the treatment achieved substantial mite reduction and remained well tolerated, meaning that no queen losses were recorded and brood disturbances were minimal. Moreover, hygienic behavior (PCC) was significantly stimulated in treated colonies, indicating that formic acid may not only control mite population but also contribute positively to colony resilience through the stimulation of natural defense mechanisms.

When compared with the results of Pietropaoli and Formato [11], who investigated different formic acid dispensers (Nassenheider Professional, MAQS, and Varterminator) alone or in combination with oxalic acid trickling, our findings show both similarities and contrasts. Their study reported variable efficacy for formic acid dispensers used alone, ranging from 49.3% with MAQS to 81.2% with Varterminator, while in combination with oxalic acid, the efficacy was boosted to 89.4–92.7%. The authors emphasize that the vapor distribution and mite mortality is significantly affected by colony strength, hive ventilation, and strip placement. However, these combined treatments were associated with notable colony losses, including reductions in adult bee populations of up to 55% and substantial brood mortality, particularly when applied under suboptimal environmental conditions.

The findings from our study indicate that Formic Pro™ achieved high *Varroa* mortality while maintaining colony integrity, consistent with results from other European and global studies. In particular, our observed efficacy rate (88.37% ± 0.23) aligns with previous trials of Smodiš Škerl et al. [39], who found that combined formic and oxalic acid treatments can sustainably suppress mite populations across seasons. This emphasizes the advantages of the controlled-release gel matrix in maintaining both efficacy and safety, as well as the importance of adhering to the prescribed doses, following the manufacturer’s instructions and temperature environmental conditions during treatment. A similar pattern emerges when our results are compared with the findings of Cabbri et al. [37], who tested 60% aqueous formic acid delivered via two evaporators (Aspro-Novar-Form and Nassenheider Professional). The mean varroacidal efficacies in their study were 81.5% and 82.0%, respectively, although with high variability among colonies (in some colonies falling below 60%). Although tolerability was generally acceptable and no significant negative effects on adult bee populations or brood were observed, variability among colonies remained a concern. Moreover, while the Aspro-Novar-Form group showed slightly a higher post-treatment adult bee number compared to Nassenheider, the difference was not statistically significant. In contrast, Formic Pro™ in our trial delivered consistently high efficacy with narrow variation, exceeding the performance of both evaporator-based systems in the study of Cabbri et al. [37].

Formic acid, like other organic acids used as miticides in beekeeping, belongs to the so-called “Generally Recognized as Safe” (GRAS) substances [40], but Tihelka [25] summarized its adverse effects observed earlier. Experimental studies cited in that review showed that high concentrations of formic acid vapors can halt oxygen consumption in brood and induce cell death in larval tissues. Additional field studies reported the suppression of proteolytic resistance of the workers’ cuticle, reductions in sealed brood area, the removal of brood cells close to absorbent pads by nurse bees, and even the weakening of colony strength when high doses or unfavorable environmental conditions were present. Furthermore, Tihelka [25] summarized an earlier study finding of temporary brood disruption, particularly under a hot climate or with overdosing. Recently, in the study of Kim et al. [41], formic acid showed the greatest risk of all tested miticides, synthetic and organic, to nurse bees. In contrast, our results with Formic Pro™ showed no such pronounced side effects; no queen mortality occurred, and observed brood effects were only minor. This may be attributed to the standardized formulation and controlled release of the active substance, as well as favorable climatic conditions during treatment in our study. Additionally, the results of our investigation indicate a very good efficacy of the applied formulation of formic acid in conditions where bee brood and intensive honey bee foraging were present. This highlights the importance of careful timing and precise dosing of the product in order to minimize collateral damage to the honey bee colonies.

An additional important dimension is the long-term sustainability of *Varroa* control. Synthetic acaricides (e. g. amitraz, fluvalinate, and coumaphos), while initially highly effective, have been repeatedly documented to induce resistance in *Varroa destructor* with excessive use [8,42,43]. The residues of these “hard acaricides” may accumulate in wax, pollen, and honey, presenting a risk for colony health and product quality [44,45]. In his review, Tihelka [25] summarized that sublethal exposure to these compounds can compromise brood survival, queen reproductive potential, drone fertility, and even adult bee immunity, thereby weakening colony resilience. Conversely, formic acid is less likely to induce resistance because of its natural occurrence in honey and its unique mode of action, which allows penetration into capped brood cells where mites reproduce [42,46,47]. This property provides a major advantage over synthetic acaricides, whose action is often limited to phoretic mites. This was clearly demonstrated in trials by Genath et al. [10], where transcriptomic profiling revealed honey bees’ innate detoxification responses to formic exposure, indicating both resilience and sensitivity thresholds.

The efficacy of Formic Pro™ recorded in our trial (88.37 ± 0.23%) was slightly lower but comparable to the results obtained by Hendriksma et al. [38], who reported 96.0 ± 1.0% efficacy for the solid matrix formulation and 95.4 ± 2.0% for the liquid Formivar 60 treatment under field conditions. The difference in efficacy between these two studies can likely be attributed to methodological and environmental factors. Hendriksma et al. [38] conducted their experiment during late summer–early autumn. Their hive type (“Dutch simplex”) was 22% smaller than the Langstroth hives used in our experiment, potentially leading to higher formic acid vapor concentrations and thereby increased acaricidal impact. A striking contrast between the two studies lies in the observed side effects. Hendriksma et al. [38] reported 1.6 times higher brood loss and approximately 30% higher queen loss in formic acid-treated colonies compared to controls, with several colonies showing stress behaviors such as bearding and absconding. In our study, no queen losses were recorded, brood disruption was minimal, and bearding occurred only briefly during the first day of treatment. Noted differences reflect variations in treatment intensity, hive volumes, ambient conditions, and colony strength, which all can influence the dynamics of formic acid evaporation. In the study of Hendriksma et al. [38], daytime temperatures frequently exceeded 25 °C, which may have accelerated acid evaporation and contributed to side effects, while our treatment period coincided with moderate weather conditions.

Another notable divergence is that our study evaluated the impact of formic acid on hygienic behavior, a colony-level defense mechanism that was not addressed in Hendriksma et al. [38]. We found a significant stimulation of hygienic behavior (PCC increase from 96.69% to 99.01% in treated colonies), indicating that Formic Pro™ may not only reduce mite loads but also activate bees’ natural disease resistance mechanisms. This finding expands on existing knowledge by linking acaricidal treatment with behavioral resilience traits, which could have implications for integrated pest management strategies. Hendriksma et al.’s [38] study primarily focused on efficacy and brood/queen impacts, highlighting operational differences between liquid and solid application methods, but it did not investigate behavioral parameters.

The relationship between honey bees and *Varroa* mites includes multiple mechanisms (acting additively or synergistically) to achieve resistance to *Varroa* mites, i.e., for their co-existence without any acaricide treatment [48]. Those defense mechanisms in bees are complex because they include numerous physical and constitutive “lines of defense” of each individual bee (physical barriers, physiological reactions, cellular and humoral immunity, gene expression, and action of the microbiome), but also special forms of behavior (behavioral mechanisms) that are necessary for maintaining “social immunity”, i.e., the collective defense of the bee colony, which is realized through numerous activities (cooperative brood care, maintenance of nest and living space hygiene, and the so-called “antiseptic behavior”, which includes the removal of dead individuals, diseased brood, and all foreign agents, ensuring the antiseptic properties of food (honey and beebread) by injecting secretions from numerous exocrine glands into it, secreting bee venom, creating propolis, and coating all surfaces inside the hive with that antimicrobial agent) [14,49]. Of the behavioral mechanisms that enable bees to fight *Varroa* mites on their own, the most significant are hygienic behavior and grooming or mite-biting behavior [14,50,51]. We note that hygienic behavior is a naturally variable-polygenic trait [52], influenced by worker task allocation, nectar flow, brood age structure, and seasonal factors [53]. The temporary decrease we observed in the Negative control group reflects natural fluctuations rather than treatment effect, particularly since baseline hygienic behavior was exceptionally high (>95%). Formic acid may influence hygienic behavior through several possible mechanisms: modulation of chemical cues within the brood nest, mild activation of detoxification and immune-related pathways, and indirect effects on brood pheromone signaling. Transcriptomic evidence indicates that formic acid exposure triggers stress and detoxification responses in honey bees, which could prime workers for increased brood inspection and removal behavior [10].

Selection for hygienic behavior was shown to bring multiple benefits to honey bee colonies, from significantly decreased *Varroa* infestation and deforming wing virus loads at the colony level to significantly increased immunity at the individual bee level [54]. Therefore, it is very important to investigate how the treatments that are commonly used in the control of *Varroa* mites influence hygienic behavior. Until now, only Gashout et al. [20] tested both varroacides as we did, formic acid and amitraz, and neither of them affected hygienic behavior. In our study, amitraz did not affect bees’ hygienic behavior, which is consistent with previous findings [20], but formic acid significantly increased the level of hygienic behavior, contradicting the results of Gashout et al. [20] but corroborating the findings of Zakaria & Allam [55]. Among other natural compounds tested as antiparasitics for use in beekeeping, only thymol increased bees’ hygienic behavior [21], while flagellin, zymosan, chitosan, and peptidoglycan [56], as well as powdered sugar [57] did not alter the hygienic potential of bees. The potential of Formic Pro™ to significantly increase the hygienic behavior of bees is an additional factor that supports the recommendation for its use in sustainable beekeeping. This study was conducted under moderate continental late-summer climatic conditions in a single geographical region. Formic Pro^®^ performance may vary in colder or warmer climates or in seasons with minimal brood areas. Additional multi-season and multi-regional trials are needed to validate applicability of Formic Pro^®^ under diverse environmental and management conditions.

## 5. Conclusions

Our findings indicate that Formic Pro™ provides reliable control of *Varroa* infestation (88.37%) and also affects the colonies’ innate disease-resistance mechanisms, as evidenced by increased hygienic activity (from 96.69% before treatment to 99.01% after treatment) in Formic Pro™-treated colonies. Combined, these effects strengthen overall colony resilience and contribute to the sustainable health management of honey bees.

## Figures and Tables

**Table 1:** Results of a descriptive analysis of the varroacidal efficacy of the formic acid-based product (Formic Pro™), amitraz as Positive control, and naturally fallen mites in the Negative control group. N: Number of hives; $\underline{x¯}$ (%): Mean value (percentage); SD: Standard deviation; Minimum: Minimum observed value; Maximum: Maximum observed value.

Groups	*n*	x¯ (%)	SD	Minimum	Maximum
Formic Pro	30	88.37	0.23	88.05	88.76
Positive control	15	94.30	0.95	93.06	95.36
Negative control	15	4.50	0.95	3.64	6.94

**Table 2:** Basic statistical parameters of hygienic behavior per group before and after the 15-day treatment period. N: Number of hives; x¯ PCC: Mean value-percent of cleaned cells; SD: Standard deviation; Minimum: Minimum observed value; Maximum: Maximum observed value.

Groups	Time-Points of Hygienic Behavior Evaluation	*n*	x¯ PCC	SD	Minimum	Maximum
Formic Pro	Before treatment	30	96.69	1.10	95.32	98.70
	After treatment	30	99.01	0.57	97.52	99.45
Positive control	Before treatment	15	97.57	0.53	96.83	98.21
	After treatment	15	97.58	0.46	97.04	98.10
Negative control	Before treatment	15	98.10	0.58	97.52	99.03
	After treatment	15	90.72	2.49	86.36	92.70

## Data Availability

The original contributions presented in this study are included in the article. Further inquiries can be directed to the corresponding author.

## Supplementary Materials

The following supporting information can be downloaded at: https://www.mdpi.com/article/10.3390/insects16121236/s1, Table S1: Daily and total mite fall in treatment groups.
