# Supplementary material for: Formic Acid-Based Preparation in Varroa destructor Control and Its Effects on Hygienic Behavior of Apis mellifera
Source: Insects. 2025 Dec 6;16(12):1236. doi: 10.3390/insects16121236 (PMC12734014; doi:10.3390/insects16121236)
Supplement: Supplementary file 1 [file insects-16-01236-s001.zip › insects-3969980-supplementary.pdf]

Supplementary table 1. Daily and total mite fall in treatment groups.

Acaricide	Average mite fall per hive		Average mite fall per hive in Follow-up treatment	Average mite fall per hive in total
	Treatment period	Daily		
Formic Pro™	1061.14	75.8	139.67	1200.81
Amitraz	1250	89.28	75.56	1325.56
